# Validation of the Italian version of the In-Person Telephone Interview for Cognitive Status (IP-TICS)

**DOI:** 10.1007/s10072-026-09201-2

**Published:** 2026-07-21

**Authors:** Edoardo Nicolò Aiello, Elena Guidetti, Giulia De Luca, Francesca Frisco, Beatrice Curti, Arianna Moreschi, Lorenzo Esposti, Sara Casartelli, Chiara Curatoli, Alice Zanin, Elisa Camporeale, Martina Andrea Sirtori, Federico Verde, Vincenzo Silani, Nicola Ticozzi, Barbara Poletti, Nadia Bolognini

**Affiliations:** 1 Department of Neurology and Laboratory of Neuroscience, IRCCS Istituto Auxologico Italiano, Milano, Italy; 2 Department of Psychology, University of Milano-Bicocca, Milano, Italy; 3 Department of Neurosurgery, IRCCS Humanitas Research Hospital, Rozzano, Milan, Italy; 4 Department of Pathophysiology and Transplantation, ″Dino Ferrari″ Center, Università degli Studi di Milano, Milano, Italy; 5 Department of Oncology and Hemato-Oncology, Università degli Studi di Milano, Milano, Italy; 6 Laboratory of Neuropsychology, Department of Neurorehabilitation Sciences, IRCCS Istituto Auxologico Italiano, Milano, Italy

**Keywords:** Telephone Interview for Cognitive Status, Cognitive screening, Tele-neuropsychology, Mini-Mental State Examination, Montreal Cognitive Assessment

## Abstract

**Background:**

This study validated the Italian version of the In-Person Telephone Interview for Cognitive Status (IP-TICS).

**Methods:**

468 healthy Italian individuals (176 males; age: 57.5 ± 15.4, education: 13.2 ± 3.7) completed the TICS, IP-TICS, MMSE and MoCA. Correlational analyses explored the construct validity of the IP-TICS against the TICS, MMSE and MoCA. Hierarchical Confirmatory Factor Analyses tested the configural, metric and scalar invariance between the TICS and IP-TICS. Statistical equivalence was assessed *via* a two one-sided test (TOST) procedure. Equating norms were derived *via* log-linear smoothing equipercentile equating (LSEE) analyses, with TOST procedures assessing equivalence between empirical and LSEE-derived scores.

**Results:**

The IP-TICS converged with the TICS, MMSE and MoCA, showing configural and metric invariance and equivalence with the TICS. Empirical and LSEE-derived scores were statistically equivalent.

**Discussion:**

Findings support the convergence, equivalence and invariance between the TICS and the IP-TICS. The IP-TICS converges with established in-person screeners.

**Supplementary Information:**

The online version contains supplementary material available at 10.1007/s10072-026-09201-2.

## Introduction

Telephone-based cognitive screening (TBCS) tests represent a flexible and acceptable alternative to deliver first-level cognitive assessments whenever topographical, logistic, and socio-economic barriers prevent examinees from accessing in-person facilities, therefore also allowing for a greater continuity of care in longitudinal scenarios [1, 2]. These instruments also serve research purposes, as facilitating the implementation and completion of large-scale, decentralized both observational and interventional studies [3]. When developing TBCS tools, it is crucial to establish whether they assess cognitive constructs comparable to traditional face-to-face measures, thereby ensuring that both administration modalities can be validly interchangeable [4, 5]. One approach to address this issue is test equating, which generates cross-walks between telephone- and in-person-based measures to support score comparability across formats [6, 7], being particularly useful when the two measures at hand are inherently different (e.g., in terms of items and range) [6]. Alternatively, evidence can be provided that the same instrument can be administered across modalities while minimizing format-related adaptations [8]. This is, for instance, the case of the recently standardized Global Examination of Mental State [9], a novel cognitive screener for which a telephone-based counterpart has been developed [10] that features the same range and includes similar items.

Unsurprisingly, efforts devoted to these goals also targeted the Telephone Interview for Cognitive Status (TICS) [11] – which arguably represents the most widespread TBCS test for global cognition, whose clinimetric soundness and optimal usability have been thoroughly established [1–3]. In fact, several algorithms have been developed worldwide that convert TICS scores to those on common in-person screening tests – such as the Mini-Mental State Examination (MMSE) [12] –, and vice-versa [6].

In this respect, and with specific regard to the Italian scenario, a key contribution lies in the 25-plus-year-old report by Ferrucci et al. [13], who explored whether the TICS, if administered face-to-face, could represent a feasible alternative to the MMSE. Within this report, the “In-Person TICS” (IP-TICS), which retained the content and scoring structure of the original TICS, showed excellent test-retest reliability, strong convergence with the MMSE, and comparable diagnostic accuracy for dementia [13]. Although Ferrucci et al. [13] originally proposed the IP-TICS as an alternative to the MMSE for individuals with sensory or motor limitations, their work also provided a proof of concept for the use of the TICS in face-to-face settings. Thus, a further, relevant follow-up to Ferrucci et al.’s [13] study would be represented by a full validation of the IP-TICS, as their report preceded the availability of the official Italian version of the TICS in 2021 [14, 15], addressed a relatively small (*N* = 104) and heterogeneous sample – including both patients with dementia (~ 40%) and healthy individuals – and did not directly compare the measurement properties of the IP-TICS with those of its original format. For such an investigation to be run properly, some key points should be considered. First, the convergence between the TICS and the IP-TICS should be established, and the measurement invariance across the two modalities demonstrated. In this regard, a further *desideratum* would be to determine the statistical equivalence between the TICS and the IP-TICS. Finally, it should be ascertained that the IP-TICS taps into the same cognitive construct captured by traditional in-person screening tests for global cognition, such as the MMSE and the Montreal Cognitive Assessment (MoCA) [16] – as recently demonstrated in respect to the TICS [17].

Beyond methodological considerations, validating an in-person adaptation of the TICS addresses a practical clinical need by determining whether the same cognitive screener can be reliably administered both over the telephone and *de visu*. Additionally, although TBCS procedures provide a practical alternative when face-to-face assessments are not feasible, routine clinical practice often includes scenarios in which traditional in-person screeners may not fully address assessment needs. As suggested by Ferrucci et al. [13]., a validated version of the IP-TICS may therefore represent a useful alternative when standard screeners such as the MMSE and MoCA are difficult to administer because of visual or motor limitations, bedside or isolation settings, or other contextual constraints affecting visuospatial or manual tasks. In these circumstances, preserving the reduced visuospatial and motor demands of the original TICS while maintaining compatibility with telephone-based administrations may facilitate continuity and flexibility in cognitive screening, including longitudinal pathways alternating telephone and in-person assessments.

Given the above premises, this study aimed at validating an Italian version of the IP-TICS by testing its convergence, measurement invariance and equivalence with the TICS, and exploring its association with widespread in-person cognitive screening test – i.e., the MMSE and the MoCA. To further support the usability of the IP-TICS, an additional scope of this report was to derive equating norms to convert the TICS to the IP-TICS, and vice-versa.

## Methods

### Participants

A total of 481 healthy Italian individuals were initially recruited between 2022 and 2024 in accordance with a non-probability sampling procedure, namely *via* Authors’ personal acquaintances and cognitive screening campaigns carried out at our institution (details blinded for peer review). Exclusion criteria included: (1) formally diagnosed neurological or psychiatric disorders; (2) history of neurodevelopmental disorders; (3) severe or uncompensated medical conditions; (4) ongoing treatment with psychotropic medications; (5) severe and/or uncorrected hearing and/or visual defects. Additionally, participants were screened *via* the MMSE [18], and those obtaining a below-cutoff age- and education-adjusted score were further excluded to rule out sub-clinical, undetected cognitive deficits.

As a result, the final sample included 468 participants, whose demographic stratification is reported in Supplementary Table 1.

The study was approved by the institutional ethics committee (details blinded for peer review) and all participants provided written informed consent.

### Materials

All participants were administered the Italian versions of the TICS [14, 15], the MMSE [18], the MoCA [19] the IP-TICS [13–15]. The Italian version of the TICS ranges from 1 to 41 and assesses *Orientation* (TICS-O, *range* = 0–12), *Language* (TICS-L, *range* = 1–8), *Memory* (TICS-M, *range* = 0–12) and A*ttention and Executive Functioning* (TICS-AEF, *range* = 0–9). The additional *Delayed Recall* task (TICS-DR; *range* = 0–10) was also administered to compute a further total score, the “TICS_&_DR” (*range* = 1–51). The IP-TICS mirrors the TICS in terms of item groupings and total/subscale-level ranges, with the following, minor procedural adjustments being made: (1) the task assessing comprehension by tapping on the phone was modified so that the examinee was instructed to tap on the table instead – as in Ferrucci et al. [13].; (2) spatial orientation items requiring to provide highly specific information (e.g., street number) were substituted with more general questions (e.g., the Country where the examinee was in that moment). We deemed this last adaptation as necessary since the IP-TICS was intended to be administered in-person, and not at the examinee’s place – as is typically the case for the TICS. In fact, requesting the examinee to know the details of the address of a place that is likely new to him/her (e.g.¸ the clinic) would have led to an underestimation of his/her spatial orientation skills.

MoCA items were grouped in accordance with Aiello et al.’s [19] subdivision, and more specifically: MoCA - *Visuo-Spatial* (MoCA-VS; *range* = 0–4), *Executive Functioning* (MoCA-EF; *range* = 0–4), *Language* (MoCA-L; *range* = 0–5), *Attention* (MoCA-A; *range* = 0–6), *Memory* (MoCA-M; *range* = 0–5) and *Orientation* (MoCA-O; *range* = 0–6). As to the MMSE, the following sub-scores were computed (as detailed elsewhere [17]): *Orientation* (MMSE-O; *range* = 0–10); *Attention* (MMSE-A, *range* = 0–5); *Memory* (MMSE-M; *range* = 0–6); *Language* (MMSE-L; *range* = 0–8). The *Constructional praxis* item was left out of these groupings and thus accounted for only within its total score.

### Procedure

Data collection was conducted by licensed psychologists and psychology trainees that underwent an extensive training focused on standardized test administration procedures. Participants underwent both in-person and telephone-based assessments. MMSE, MoCA, and IP-TICS were administered in person, while TICS was administered by telephone. To take under control potential carry-over effects, we randomly selected 158 participants that took the TICS first and were then administered the in-person screeners, with remaining participant (*N* = 310) participants completing the in-person session before the TICS. The interval between the two sessions was approximately 5–7 days.

The TICS was administered in accordance with a previously established protocol [17]. Participants took the test while being at their place. A third person was required to witness to the administration of the TICS so that he/she could ensure that neither cues/facilitating items (e.g., a calendar; paper and pencil; etc.) nor distractions were present within the testing environment. This third party was also asked to confirm (or not) the correctness of examinees’ responses to spatial orientation items. A brief sound-check was also conducted to verify that the quality of the audio was acceptable from both the examinee’s and the examiner’s standpoints.

In-person sessions followed this fixed administration sequence: MMSE, MoCA, and IP-TICS. To avoid redundant testing, overlapping items across these three tests (namely, the “Serial Sevens” task and some questions on spatial and temporal orientation) were only re-administered if the participant failed the preceding attempt. In alignment with a previously established procedure [17, 20], any item successfully completed in an earlier test was automatically scored as correct for all subsequent assessments.

### Statistical analysis

#### Background analyses

Normality checks were run on each cognitive measure by addressing skewness values – judged as indexing a violation of the assumption if ≥|1 [21] – and by visually inspecting histograms and Q-Q plots for abnormalities.

To explore whether the administration order – i.e., telephone-based first vs. in-person first – affected examinee’s performances on the IP-TICS/IP-TICS_&_DR, independent-sample *t-*tests were run on both IP-TICS and IP-TICS_&_DR scores by addressing the abovementioned classification as the factor.

#### Convergent validity

Given that the vast majority of cognitive scores did not distribute normally, the convergent validity of the IP-TICS/IP-TICS_&_DR against the TICS/TICS_&_DR on one hand and the MMSE and MoCA on the other was tested *via* Spearman’s coefficients. More specifically, correlations were run both by addressing total scores on these tests and subscale-level scores. The MMSE-O was left out of subscale-level correlational analyses given that the vast majority of its items overlapped with those of the IP-TICS-O. Whenever appropriate, Bonferroni’s correction was applied to the significance threshold in order not to incur in an inflation of Type-I error rates – with α_adjusted_ being computed as 0.05/*k*, with *k* being the number of correlations run. The magnitude of each Spearman coefficient was interpreted according to Cohen’s [22] benchmarks – i.e., *r*_*s*_≤0.30 → small; 0.30 < *r*_*s*_≤0.50 → medium; *r*_*s*_≥0.50 → large. Following Hobart’s [23] guidelines, which indicate that validity analyses should require at least 80 observations, the current sample size was considered as appropriate to this aim.

#### Measurement invariance

The measurement invariance between the TICS and IP-TICS was assessed by entering each sub-score – i.e., TICS-/IP-TICS-O, -L, -M and -AEF – in a hierarchical series of Confirmatory Factor Analyses (CFA) for repeated measures [24] conducted by means of the *SEMLj* module in jamovi (https://semlj.github.io/) – which, in turn, implements the *lavaan* package in R [25]. The hierarchical procedure included three steps [24] – each based on the fact that the Italian TICS is underpinned by a mono-dimensional structure (“global cognition”) [14, 15]. First, we tested configural invariance, namely whether the same, mono-dimensional structure held for both the TICS and the IP-TICS. Subsequently, factor loadings were constrained to be equal across the two tests to assess whether each subscale contributed equally to the target construct regardless of the test being administered over the telephone vs. in person (metric invariance). Finally, sub-score intercepts were further constrained to be equal across the TICS and the IP-TICS to test scalar invariance – i.e., whether examinees’ performances, at the same level of the latent trait, are comparable across the two tests. Given that data were statistically dependent, residual covariances between corresponding sub-scores across the two tests were accounted for within each of these steps. Individual model fit was evaluated *via* the Comparative Fit Index (CFI), the Root Mean Square Error of Approximation (RMSEA) and the Standardized Root Mean Square Residual (SRMR) – being judged as appropriate if > 0.95, ≤0.05 and ≤ 0.07, respectively [26]. According to Chen [27], invariance was supported if the decrease in CFI values and the increase in RMSEA values between increasingly restrictive models was ≤−0.01 and ≤ 0.015, respectively. Hierarcical CFAs were also run to test the measurement invariance between the TICS_&_DR and the IP-TICS_&_DR. As reported by Kyriazos [28], at least 100 observations need to be considered to run a CFA; hence, our sample size was adequate to run these analyses.

#### Equivalence testing

The statistical equivalence between the TICS/TICS_&_DR and the IP-TICS/IP-TICS_&_DR was tested by means of Laken’s [29] two one-sided test (TOST) procedure. Such an approach allows to conclude on the statistical equivalence between two dependent means if the effect size of their difference falls within a prespecified equivalence interval (i.e., when the difference is simultaneously statistically greater than the lower bound and statistically smaller than the upper bound). TOST analyses were run via the dedicated jamovi macro relying on the R package *TOSTER* [29, 30]. Based on Lakens [29], a minimum of 44 pairs of observations are adequate for a TOST analysis when addressing a significance level α of 0.05, a power 1-β of 0.9, and equivalence bounds between − 0.5 and 0.5 (these being default values in the absence of specific hypothesis regarding the smallest meaningful effect size).

#### Equating norms

Score equating was performed using the log-linear smoothed equipercentile equating (LSEE) approach [7] through the R package *equi* [31], producing conversion tables between TICS/TICS_&_DR and IP-TICS/IP-TICS_&_DR. The LSEE method converts measures by matching their percentile ranks with a log-linear function that reduces distribution irregularities [7]. To the aim of these analyses, our sample size included a sufficiently large number of observations based on Livingston’s [32] recommendations, which suggest a sample size of at least *N* = 200 for LSEE analyses.

In accordance with previous reports on the topic [17, 33, 34], a TOST procedure for dependent measures was subsequently to validate the estimated cross-walks, namely to determine whether equated scores derived through LSEE algorithms were statistically equivalent to empirical ones. In these analyses, LSEE-derived scores were rounded up in order for them to be integer given that empirical test scores do not foresee the presence of decimals.

Analyses were performed using R 4.3.0 (R Core Team, 2023), SPSS 29 (IBM Corp., 2023) and jamovi 2.6 (The jamovi project, 2024). Three participants did not complete the *Delayed Recall* task of the IP-TICS; therefore, analyses regarding this measure and the IP-TICS_&_DR were run on 465 observations (vs. 468).

## Results

Participants’ demographic and cognitive characteristics are summarized in Table 1. Participants being tested over the telephone first reported higher (*t*(466)=−4.37; *p*<.001) IP-TICS scores when compared to those undergoing the in-person screening session first (36.25 ± 1.98 vs. 35.30 ± 2.35). The same was true for the IP-TICS_&_DR (*t*(463)=−4.19; *p*<.001; 39.77 ± 3.88 vs. 38.09 ± 4.17).

**Table 1:** Participants’ demographic and cognitive data

*N*	468
**Age (years)**	57.5 ± 15.4 (20–94)
**Sex (male/female)**	176/292
**Education (years)**	13.2 ± 3.7 (5–25)
**MMSE**	29.3 ± 0.9 (26–30)
**MoCA**	25.4 ± 3 (14–30)
Executive function	3.3±0.9 (0–4)
Attention	5.5±0.9 (2–6)
Language	4.8±0.5 (2–5)
Visuo-spatial abilities	3.4±0.8 (0–4)
Orientation	5.9 ± 0.2 (4–6)
Delayed Recall	2.5 ± 1.7 (0–5)
**TICS**	35.9 ± 2.4 (24–41)
Attention and Executive Functioning	8.6±0.9 (3–9)
Language	7.9±0.3 (6–8)
Memory	7.5 ± 1.9 (0–12)
Orientation	11.9±0.3 (9–12)
Delayed Recall	4.1 ± 2.2 (0–10)
**TICS**_**&**_ **DR**	40 ± 4.1 (24–51)
**IP-TICS**	35.6 ± 2.3 (25–41)
Attention and Executive Functioning	8.8 ± 0.8 (4–9)
Language	7.9±0.3 (6–8)
Memory	7.1 ± 1.8 (1–12)
Orientation	11.9±0.4 (9–12)
Delayed Recall	3.1 ± 2.2 (0–10)
**IPTICS**_**&**_ **DR**	38.7 ± 4.1 (25–51)

IP-TICS = In-Person Telephone Interview for Cognitive Status; IPTICS&DR = In-Person Telephone Interview for Cognitive Status with Delayed Recall; MoCA= Montreal Cognitive Assessment; MMSE= Mini-Mental State Examination; TICS= Telephone Interview for Cognitive Status; TICS& DR= Telephone Interview for Cognitive Status with Delayed Recall. Continuous outcomes are shown as *M* ± *SD* (*range*), whilst categorical ones as frequencies and/or percentages

### Convergent validity

The IP-TICS showed a large correlation with the TICS (*r*_*s*_(468)=0.52; *p*<.001), with the same being true for the association between the TICS_&_DR and the IP-TICS_&_DR (*r*_*s*_(468)=0.56; *p*<.001). Consistently, at α_adjusted_ = 0.025, the IP-TICS and the IP-TICS_&_DR showed medium-to-large association with both the MMSE (IP-TICS: *r*_*s*_(468)=0.34; *p*<.001; IP-TICS_&_DR: *r*_*s*_(465)=0.33; *p*<.001) and the MoCA (IP-TICS: *r*_*s*_(468)=0.48; *p*<.001; IP-TICS_&_DR: *r*_*s*_(465)=0.53; *p*<.001).

Table 2 shows the results of subscale-level convergent validity testing analyses between the TICS and the IP-TICS. Overall, at α_adjusted_ = 0.002, each IP-TICS sub-scale showed medium-to-large correlations with its TICS counterpart, with the exception of the *Orientation* one. In addition, and expectedly, the IP-TICS-M converged with the TICS-DR and, consistently, the IP-TICS-DR converged with the TICS-M. Two further association survived Bonferroni’s correction – albeit being small in size: that between the IP-TICS-AEF and the TICS-M and that between the IP-TICS-L and the TICS-DR.

**Table 2:** Spearman coefficients for the association between TICS and IP-TICS subscales

	TICS-O	TICS-M	TICS-AEF	TICS-L	TICS-DR
**IP-TICS-O**	0.13	0.14	0.10	0.04	0.07
**IP-TICS-M**	0.14	.47*	0.16*	0.09	0.41*
**IP-TICS-AEF**	0.04	0.14	0.43*	0.05	0.14
**IP-TICS-L**	0.10	0.14	0.13	0.43*	0.17*
**IP-TICS-DR**	0.12	0.46*	0.13	0.05	0.53*

IP-TICS-O = In-Person Telephone Interview for Cognitive Status-Orientation; IP-TICS-M = In-Person Telephone Interview for Cognitive Status-Memory; IP-TICS-AEF = In-Person Telephone Interview for Cognitive Status-Attention and Executive Function; IP-TICS-L = In-Person Telephone Interview for Cognitive Status-Language; IP-TICS-DR = In-Person Telephone Interview for Cognitive Status-Delayed Recall; TICS-O= Telephone Interview for Cognitive Status-Orientation; TICS-M= Telephone Interview for Cognitive Status-Memory; TICS-AEF= Telephone Interview for Cognitive Status-Attention and Executive Function; TICS-L= Telephone Interview for Cognitive Status-Language; TICS**-**DR= Telephone Interview for Cognitive Status-Delayed Recall. *Significant coefficient at α_adjusted_ = 0.05/*k*=0.05/25=0.002

Subscale-level correlation coefficients between the IP-TICS and established in-person screeners (MMSE; MoCA) are displayed in Table 3 (α_adjusted_ = 0.001). The IP-TICS-O expectedly showed a strong association with the MoCA-O only. The IP-TICS-M showed small-to-medium associations with the MMSE-M and all MoCA subscales except for the MoCA-O. The same pattern was detected as to the IP-TICS-DR – with the sole difference lying in the lack of association between this measure and the MoCA-VS. With regard to the IP-TICS-AEF, small-to-medium correlations were detected with the MMSE-A, the MMSE-L and MoCA-A. By contrast, no association involving the IP-TICS-L survived Bonferroni’s correction.

**Table 3:** Spearman’s coefficients for the association between IP-TICS and MMSE/MoCA subscales

	IP-TICS-O	IP-TICS-M	IP-TICS-AEF	IP-TICS-L	IP-TICS_−_DR
**MMSE-M**	0.06	0.20*	0.09	0.09	0.23*
**MMSE-A**	− 0.04	0.10	0.24*	0.10	0.14
**MMSE-L**	0.02	0.13	0.18*	0.03	0.06
**MoCA-VS**	0.06	0.16*	0.10	0.14	0.14
**MoCA-EF**	0.09	0.25*	0.14	0.12	0.24*
**MoCA-L**	0.08	0.22*	0.14	0.11	0.18*
**MoCA-A**	0.08	0.33*	0.32*	0.11	0.31*
**MoCA-DR**	0.13	0.34*	0.14	0.10	0.43*
**MoCA-O**	0.65*	0.08	0.03	0.08	0.09

IP-TICS-O = In-Person Telephone Interview for Cognitive Status-Orientation; IP-TICS-M = In-Person Telephone Interview for Cognitive Status-Memory; IP-TICS-AEF = In-Person Telephone Interview for Cognitive Status-Attention and Executive Function; IP-TICS-L = In-Person Telephone Interview for Cognitive Status-Language; IP-TICS-DR = In-Person Telephone Interview for Cognitive Status-Delayed Recall; MMSE-M= Mini-Mental State Examination-Memory; MMSE-A= Mini-Mental State Examination-Attention; MMSE-L= Mini-Mental State Examination-Language; MoCA-VS= Montreal Cognitive Assessment-Visuo-Spatial abilities; MoCA-EF= Montreal Cognitive Assessment-Executive Function; MoCA-L= Montreal Cognitive Assessment-Language; MoCA-DR= Montreal Cognitive Assessment-Delayed Recall; MoCA-O= Montreal Cognitive Assessment-Orientation. *Significant coefficient at α_adjusted_ = 0.05/*k*=0.05/45=0.001

### Measurement invariance

The results of hierarchical CFAs aimed at assessing the measurement invariance between the TICS and the IP-TICS, both with and without the *Delayed Recall* task, are reported in Table 4. As to the TICS-vs.-IP-TICS comparison, the configural model supported a stable, single-factor structure across the two tests. Full metric invariance was also satisfied, with both individual fit indices and changes in CFI and RMSEA falling within acceptable ranges. However, scalar invariance happened to be rejected, with *post-hoc* constraint score tests revealing that this was driven by significant differences in the intercepts of the *Attention and Executive Functioning* (χ^2^(1) = 32.42; *p*<.001), *Memory* (χ^2^(1) = 29.46; *p*<.001) and *Language* (χ^2^(1) = 4.17; *p*=.041) subscales. When adding to the model the *Delayed Recall* task (i.e., TICS_&_DR-vs.-IP-TICS_&_DR comparison), the configural model still showed an adequate fit to the data, with full metric invariance being again supported as well. Moreover, and similarly to the first model, scalar invariance was not achieved – with *post-hoc* constraint score tests revealing that this was due to significant discrepancies in the intercepts of the *Delayed Recall* (χ^2^(1) = 65.17; *p*<.001) and *Attention and Executive Functioning* (χ^2^(1) = 41.13; *p*<.001) subscales.

**Table 4:** Results of hierarchical CFAs testing the measurement invariance between the TICS and the IP-TICS (both with and without the *Delayed Recall* task)

		χ^2^	df	*p*	CFI	RMSEA [95% CI]	SRMR	ΔCFI	ΔRMSEA	Invariance
**TICS**	Configural	27.75	15	0.023	0.976	0.043 [0.016, 0.067]	0.031	-	-	Supported
	Metric	31.70	18	0.024	0.974	0.040 [0.015, 0.063]	0.034	0.002	0.003	Supported
	Scalar	101.33	22	< 0.001	0.851	0.088 [0.071, 0.105]	0.053	0.123	0.048	Rjected
**IP-TICS**	Configural	68.74	29	< 0.001	0.971	0.054 [0.038, 0.071]	0.049	-	-	Supported
	Metric	74.92	33	< 0.001	0.969	0.052 [0.037, 0.068]	0.051	0.002	0.002	Supported
	Scalar	225.60	38	< 0.001	0.863	0.103 [0.090, 0.116]	0.080	0.106	0.051	Rejected

CFA=Confirmatory Factor Analysis; TICS=Telephone Interview for Cognitive Status; IP-TICS = In-Person Telephone Interview for Cognitive Status; CFI=Comparative Fit Index; RMSEA=Root Mean Square Error of Approximation; SRMR=Standardized Root Mean Square Residual

### Equivalence testing

The results of the TOST procedure for testing the equivalence between the TICS/TICS_&_DR and the IP-TICS/IP-TICS_&_DR are shown in Table 5. In spite of a significant between-group difference as yielded by the dependent-sample *t-*test, the TICS proved to be equivalent to the IP-TICS – with both the lower and upper equivalence bounds falling within the predefined limits. At variance, equivalence between the TICS_&_DR and the IP-TICS_&_DR was rejected – with scores on the former test being higher than those on the latter.

**Table 5:** Results of the TOST procedure for testing the equivalence between TICS/TICS_&_DR and IP-TICS/IP-TICS_&_DR

TICS		IP-TICS		t	*p*	Equivalence
TICS	vs.	IP-TICS	*t*-test	3.3	**< 0.001**	Supported
35.9 ± 2.38		35.6 ± 2.28	Upper EB	8.6	**< 0.001**	
			Lower EB	1.8	**0.033**	
TICS_&_DR	vs.	IP-TICS_&_DR	*t*-test	−7.9	**< 0.001**	Rejected
40 ± 4.13		38.7 ± 4.14	Upper EB	10.8	**< 0.001**	
			Lower EB	−4.9	1.00	

IP-TICS = In-Person Telephone Interview for Cognitive Status; IP-TICS_&_DR = In-Person Telephone Interview for Cognitive Status with Delayed Recall; TICS= Telephone Interview for Cognitive Status; TICS_**&**_ DR= Telephone Interview for Cognitive Status with Delayed Recall; EB=equivalent bound. Continuous outcomes are shown as *M* ± *SD* (*range*)

### Equating norms

Table 6 reports LSEE-based crosswalks to convert TICS/TICS_&_DR scores to those on the IP-TICS/IP-TICS_&_DR, and vice-versa. Conversions could not be derived for TICS/TICS_&_DR and IP-TICS/IP-TICS_&_DR scores below 24 and 25, respectively, since such lowermost scores were not represented within the present sample. For the same reason, no counterparts were found for TICS/TICS_&_DR scores equal to 25, 26 and 28, as well as for IP-TICS/IP-TICS_&_DR scores equal to 26 and 27. Within TICS-to-IP-TICS and IP-TICS-to-TICS crosswalks, both the source and the equated score were mostly identical. Similarly, as to TICS_&_DR-to-IP-TICS_&_DR and IP-TICS_&_DR-to-TICS_&_DR crosswalks, the raw difference between source and equated scores were minimal and typically ranged from 0 to |2|.

**Table 6:** LSEE-derived tables to convert TICS/TICS_&_DR scores to those on the IP-TICS/IP-TICS_&_DR, and vice-versa

TICS to IP-TICS			IP-TICS to TICS			TICS_&_DR to IP-TICS_&_DR			IP-TICS_&_DR to TICS_&_DR	
TICS	IP-TICS		IP-TICS	TICS		TICS_&_DR	IP-TICS_&_DR		IP-TICS_&_DR	TICS_&_DR
24	25		25	25		24	26		25	24
25	-		26	-		25	-		26	-
26	-		27	-		26	-		27	-
27	26		28	28		27	27		28	28
28	-		29	29		28	-		29	30
29	29		30	30		29	28		30	31
30	30		31	31		30	29		31	32
31	31		32	32		31	30		32	34
32	32		33	33		32	30		33	35
33	33		34	34		33	31		34	36
34	34		35	35		34	32		35	36
35	35		36	36		35	33		36	37
36	36		37	37		36	34		37	38
37	37		38	39		37	36		38	39
38	38		39	40		38	37		39	40
39	39		40	41		39	38		40	41
40	40		41	41		40	39		41	42
41	41					41	40		42	43
						42	41		43	44
						43	42		44	45
						44	43		45	46
						45	44		46	48
						46	45		47	49
						47	46		48	50
						48	46		49	50
						49	47		51	51
						50	48			
						51	51			

IP-TICS = In-Person Telephone Interview for Cognitive Status; IP-TICS_&_DR = In-Person Telephone Interview for Cognitive Status with Delayed Recall; TICS= Telephone Interview for Cognitive Status; TICS_**&**_ DR=Telephone Interview for Cognitive Status with Delayed Recall

The results of TOST analyses aimed at validating the aforementioned crosswalks are reported in Table 7. Equivalence between empirical and LSEE-derived scores was supported for each cross-walk.

**Table 7:** TOST procedure for the comparison between empirical and LSEE-derived scores

Empirical score		Derived score		t	*p*	Equivalence
TICS	vs.	TICS derived from IP-TICS	*t*-test	1.4	0.152	Supported
35.9 ± 2.38		35.8 ± 2.50	Upper EB	3.4	**< 0.001**	
			Lower EB	6.3	**< 0.001**	
IP-TICS	vs.	IP-TICS derived from TICS	*t*-test	3.3	**0.001**	Supported
35.6 ± 2.28		35.9 ± 2.40	Upper EB	8.5	**< 0.001**	
			Lower EB	1.9	**0.030**	
TICS_&_DR	vs.	TICS_&_DR derived from IP-TICS_&_DR	*t*-test	1.1	0.276	Supported
40 ± 4.13		39.8 ± 4.11	Upper EB	4.0	**< 0.001**	
			Lower EB	1.9	**0.032**	
IP-TICS_&_DR	vs.	IP-TICS_&_DR derived from TICS_&_DR	*t*-test	1.0	0.323	Supported
38.7 ± 4.14		38.8.±4.28	Upper EB	1.9	**0.030**	
			Lower EB	3.9	**< 0.001**	

IP-TICS = In-Person Telephone Interview for Cognitive Status; IPTICS_&_DR = In-Person Telephone Interview for Cognitive Status with Delayed Recall; TICS= Telephone Interview for Cognitive Status; TICS_**&**_ DR= Telephone Interview for Cognitive Status with Delayed Recall; EB=equivalent bound. Continuous outcomes are shown as *M* ± *SD* (*range*)

## Discussion

The present study provides the Italian validation of the in-person version of the TICS to be administered face-to-face (i.e., the IP-TICS), supporting its use in clinical and research settings where assessment modality may vary or standard screening procedures are difficult to implement. More specifically, this study supports the convergence, equivalence and configural and metric invariance between the IP-TICS and the TICS, also providing evidence for the construct validity of the IP-TICS against two established in-person cognitive screening tests: the MMSE and the MoCA. In addition, we deliver LSEE-based, straightforward conversion table to estimate TICS scores from IP-TICS ones, and vice-versa.

### Convergence, invariance and equivalence between the TICS and the IP-TICS

A clinically relevant question concerns whether the IP-TICS and the telephone-based TICS provide comparable measures of cognitive functioning, thus allowing clinicians and researchers to interpret scores consistently. We approached this matter by exploring the convergent validity, measurement invariance and statistical equivalence between the two measures. As to convergent validity analyses, the IP-TICS proved to be strongly associated with the TICS – this being true also when including the *Delayed Recall* task. Notably, moderate-to-strong correlations were also found at the subscale level – with each IP-TICS sub-score, except for the IP-TICS-O, converging with its telephone-based counterparts. These findings mostly align with those yielding from measurement invariance testing. In fact, full configural and metric invariance between the TICS/TICS_&_DR and the IP-TICS/IP-TICS_&_DR was supported, this suggesting that: (1) the mono-dimensional structure of the TICS is stable regardless of whether it is administered over the telephone or in person and (2) each of its sub-scale contributes equally to the target construct (i.e., “global cognition”) across the two formats. Taken together, these findings avail the notion that the IP-TICS/IP-TICS_&_DR is overall comparable to the TICS/TICS_&_DR in terms of what is being measured – namely, global cognition/overall cognitive efficiency.

At the same time, full scalar invariance between the TICS and the IP-TICS (as well as between the TICS_&_DR and the IP-TICS_&_DR) was not met, suggesting that administration modality may influence performance on specific subdomains despite overall comparability between formats. When looking at *post-hoc* constraint score tests, *Attention and Executive Functioning*, *Memory* and – although marginally – *Language* subscales contributed to the lack of scalar invariance within the TICS-vs.-IP-TICS comparison, while this was true for the *Attention and Executive Functioning* and *Delayed Recall* subscales when comparing the TICS_&_DR to the IP-TICS_&_DR. Such findings hint at the fact that, at least when dealing with attentional-executive and memory sub-scales, construct-irrelevant confounders related to the different modality of administration account for potential discrepancies in test scores between the TICS/TICS_&_DR and the IP-TICS/IP-TICS_&_DR.

While seemingly contradictory, these findings are mostly in line with the results from the TOST procedure. Indeed, although the IP-TICS was found to be statistically equivalent to the TICS, this equivalence did not extend to the TICS_&_DR-vs.-IP-TICS_&_DR comparison. Furthermore, despite the effect size of the difference between IP-TICS and TICS scores falling within the predefined equivalence bounds, the dependent-sample *t*-test still revealed a significant discrepancy between the two measures. It might thus be the case that the significant *t*-test result at the total score level is driven by the subscale-level intercept differences identified by the scalar invariance CFA, especially within the *Attention and Executive Functioning* and *Memory* subscales. Taken together, these results suggest that, while subscale-level inconsistencies might exist between the IP-TICS and the TICS, such differences are likely to be attenuated when addressing the total score, thereby leading to the two tests being statistically equivalent at a global level. One possible interpretation is that modality-specific performance variations concerning a given subscale – e.g., *Memory* – may be counterbalanced by more stable scores on tasks enclosed within domains that are less subject to variability – e.g., *Orientation* –, thereby preserving the equivalence of total scores across the two formats. From another perspective, it could be argued that such total-score equivalence might also reflect the masking of localized mode effects rather than absolute construct equivalence across all subdomains – a possibility that warrants further investigations.

Notably, this pattern did not extend to the versions of the test also including the *Delayed Recall*, suggesting that modality-related effects may become more evident when delayed memory performance is considered. In this last case, the subscale-level discrepancies are likely to be too large to be diluted within the total score. In the case of our cohort, participants happened to score higher on the *Delayed Recall* task when this was administered over the telephone. As previously suggested [5], it is possible that the telephone *medium* reduces performance anxiety related to highly demanding tasks – such as those requiring the delayed retrieval of verbal items [35] – due to the lack of physical examiner-examinee interactions and the fact that the examinee is tested in a familiar environment (i.e., his/her place).

It is interesting to note that most of the abovementioned findings closely align with a recent, large-scale study carried out by Smith et al. [5], where the comparability between telephone-based and in-person performances on some tasks drawn from the TICS was assessed in a population-based sample of 6,825 individuals aged ≥ 65 years. Although these Authors^5^ embraced an independent-sample study design (i.e., some individuals taking the tests over the telephone and some others in person), they likewise found that, despite cross-modality configural, metric and scalar invariance being met, significant discrepancies emerged in the intercepts of memory and attention measures.

### Crosswalks between the IP-TICS and the TICS

A key contribution of our study lies in the provision of equating norms to convert IP-TICS/IP-TICS_&_DR scores to TICS/TICS_&_DR ones, and vice-versa, providing clinicians with a practical tool to compare cognitive performance across telephone and face-to-face assessments. Given the mixed results of measurement invariance analyses, which do not entirely support the full comparability between the two tests, the availability of such conversion tables plays an even more important role towards clinical and research practice. In fact, these crosswalks properly allow to level out the between-modality discrepancies in test scores that have been outlined by measurement invariance and equivalence analyses. Most relevantly, these conversions happened to be supported by TOST analyses comparing empirical to LSEE-derived scores – thus providing evidence for the validity of the crosswalks themselves.

As to TICS-to-IP-TICS and IP-TICS-to-TICS conversions, it is interesting to note that source and equated scores were often identical, with the exception of lowermost values. This occurrence suggests that the lack of scalar invariance reported above might selectively apply, at least to some extent, to those examinees scoring on the lower range of the test. This hypothesis is once again supported by Smith et al.’s [5] study, which demonstrated that discrepancies between the different modalities are particularly evident in examinees with poor performances.

With regard to the crosswalks for the test formats including the *Delayed Recall* task, source and target scores were only infrequently identical, in line with both the lack of equivalence and of scalar invariance between the TICS_&_DR and the IP-TICS_&_DR.

### Construct validity of the IP-TICS against established in-person screeners

From a clinical perspective, establishing whether the IP-TICS captures cognitive constructs comparable to those assessed by widely used screening measures is essential to support its interpretability within routine assessment pathways. Thus, a further, relevant findings lie in the demonstration that the total score on the IP-TICS – both with and without the *Delayed Recall* task – moderately-to-strongly converges with widespread cognitive screening tests such as the MMSE and the MoCA. This suggests that the IP-TICS/IP-TICS_&_DR captures cognitive constructs broadly comparable to established in-person screeners. This is also consistent with a recent Italian study [17] supporting the convergent validity between TICS/TICS_&_DR scores and both the MMSE and the MoCA.

The results of correlational analyses at a total score level mostly aligned with those at the subscale level – with significant associations being found between IP-TICS sub-scores and MMSE/MoCA ones aimed at measuring similar cognitive domains/functions, with the exception of the IP-TICS-L. In fact, this last subscale showed not significant associations with MMES/MoCA sub-scores after Bonferroni’s correction. A possible explanation to this finding might lie in the relatively low variability of language measures across the three tests. This is consistent with the notion that ceiling effects are often found in healthy individuals’ scores on simple language tests [27, 36]. A further finding that is worth discussing is represented by the fact that the IP-TICS-M and the IP-TICS-DR showed a widespread pattern of associations with MMSE/MoCA sub-scores. More specifically, these two IP-TICS sub-scores not only converged with MMSE/MoCA sub-scale assessing memory, but also with those assessing executive functioning, attention, language and, with specific regard to the IP-TICS-M, visuo-spatial skills. This finding is consistent with the notion that that the performance on memory tasks enclosed within multi-component screening tests reflect an integrative process engaging multiple cognitive systems [35].

### Implications for clinical practice and research

This study adds up to and complements the growing Italian literature on TBCS tests [10, 14, 15, 37–41], and, more specifically, on the matter of the comparability between TBCS tests and in-person cognitive screeners [10, 13, 17].

First, our report delivers evidence regarding the potential application of the TICS and the IP-TICS within both clinical practice and research settings. In fact, the convergence, configural/metric invariance and equivalence between the two tests warrant that the underlying construct(s) being measured is stable across telephone and in-person modalities. The association between the IP-TICS and established in-person screening tests (MMSE; MoCA) further strengthens its validity as measure of global cognition. The availability of the present crosswalks to convert IP-TICS scores to those on the TICS – and vice-versa – closes the circle, as allowing for a proper comparability between these two tests by overcoming the lack of scalar invariance. Based on these findings, clinicians and researchers may adopt more flexible cognitive screening pathways by adapting assessment modality according to patient characteristics and contextual constraints while preserving comparability of cognitive estimates across evaluations. In particular, compatibility between the TICS and IP-TICS may be especially relevant when standard screening procedures are not feasible (e.g., bedside evaluations, individuals with visual or motor limitations, isolation settings, or longitudinal protocols alternating telephone and face-to-face assessments).

For instance, a patient can be screened at baseline *via* the IP-TICS and then followed-up with the TICS, and vice-versa, without the administration modality preventing users from properly comparing longitudinal scores. In addition, within research settings, our conversion tables could help harmonize data collected *via* different formats of the test – especially within longitudinal, multi-center investigations. Finally, as suggested by Ferrucci et al. [13], this report supports the use of the IP-TICS as a valid proxy of global cognitive functioning in contexts where standard in-person cognitive screening tests are not feasible, such as when visual, motor, or setting-related confounders interfere with the administration and/or execution of tasks requiring visual information processing (from both the examiner’s and the examinee’s standpoints) or the provision of motor responses [43]. By contrast, as to the TICS_&_DR/IP-TICS_&_DR, the lack of equivalence between the telephone-based and in-person formats – in spite of the other findings being comparable to those addressing the TICS/IP-TICS – suggests that extreme caution should be exerted when comparing the two tests if the *Delayed Recall* task is also administered. Hence, also in the light of the facts that, firstly, this last sub-score proved not to increase the degree of informativity toward examinees’ cognitive *status* within the Italian standardization of the TICS [14], secondary, the Italian version of the TICS without the *Delayed Recall* task still showed optimal clinimetric properties for the detection of mild cognitive impairment and dementia [41], we advise clinicians and researchers to focus on the “original” TICS/IP-TICS total (i.e., the one ranging 1–41) unless it is strictly relevant to their goals to additionally consider the *Delayed Recall* sub-score.

### Limitations and future perspective

This study is of course not devoid of limitations. First and foremost, our sample only included healthy individuals, this limiting the generalizability of our findings toward either cognitively unimpaired individuals or examinees with subjective/mild cognitive dysfunctions. Accordingly, these findings should be interpreted as preliminary evidence of psychometric comparability, while further validation in clinical sample is essential before adopting widespread utilization in diagnostic protocols. Such a stance is mostly evident with regard to the present equating norms – which are unlikely to be useful for patients with moderate-to-severe cognitive impairment, given that conversions for lowermost scores (i.e., ≤ 24 for the TICS/IP-TICS and ≤ 25 for the TICS_&_DR/IP-TICS_&_DR) could not be estimated by the LSEE. Hence, a key goal that should be addressed within future studies on the topic would be to replicate the present analyses in patients with brain disorders [44]. Second, this study did not focus on providing normative data for the IP-TICS. In spite of the demonstrated equivalence, we caution against the straightforward application of the existing Italian normative framework of the TICS [15] to the IP-TICS in formal clinical settings. In fact, unknown sources of systematic error variance inherent to the face-to-face modality might still influence examinees’ performances, thus potentially undermining the validity of norms delivered for a test to be administered over the telephone [45]. Nevertheless, one possibility is that clinicians may cautiously apply TICS norms to the IP-TICS for exploratory purposes, provided that the lack of IP-TICS-specific normative data is explicitly acknowledged. By contrast, for research purposes, such an application may be considered more acceptable – although, once again, with caution, meaning that the potential for modality-related measurement errors is carefully accounted for when interpreting the results. With that being said, in the light of these considerations, it would be advisable for future studies to aim at deriving Italian norms for the IP-TICS. A further issue lies in the fact that some practice effects have been detected in relation to the IP-TICS: participants who took the TICS first scored higher on the IP-TICS than those who took the latter test before the TICS. Consequently, the relatively short interval between administrations (5–7 days) may have introduced carry-over effects, potentially increasing score similarity across formats, inflating equivalence estimates and, to some extent, influencing the derived conversion norms. Similarly, associations between the IP-TICS and established in-person screening measures should be interpreted cautiously, as MMSE and MoCA were systematically administered before the IP-TICS, potentially introducing order-related influences or practice effects. Moreover, identical items overlapping across screening measures were not re-administered within the IP-TICS if previously administered. Therefore, some scores may have been influenced by earlier assessments, potentially introducing circularity and inflating associations with MMSE and MoCA. Therefore, convergent validity findings should be interpreted cautiously.

### Conclusions

This study supports the convergence, equivalence and configural and metric invariance between the TICS and the IP-TICS in an Italian population sample, offering promising evidence of their comparability across telephone and face-to-face administrations. Moreover, the IP-TICS converges with established in-person cognitive screening tests, supporting its potential utility in clinical settings where flexible assessment pathways or alternatives to standard screeners may be needed.

## Electronic Supplementary Material

Below is the link to the electronic supplementary material.

Supplementary Table 1 (DOCX 15.5 KB)

## Data Availability

Datasets associated with the present study cannot be made publicly available on ethical-legal grounds but have been stored on an online respository with restricted access (10.5281/zenodo.21361399) and can be made available upon reasonable request of interested researchers to the Corresponding Author(s), who will forward a request for a data transfer agreement to the relevant Ethical Committee. Data concerning the Telephone Interview for Cognitive Status cannot be made available due to copyright-related reasons.
