# Supplementary material for: Validation of the Italian version of the In-Person Telephone Interview for Cognitive Status (IP-TICS)
Source: Neurol Sci. 2026 Jul 21;47(8):649. doi: 10.1007/s10072-026-09201-2 (PMC13388649; doi:10.1007/s10072-026-09201-2)
Supplement: Supplementary file 1 — Supplementary Table 1 (DOCX 15.5 KB) [file 10072_2026_9201_MOESM1_ESM.docx]

**Supplementary Table 1**. Sample stratification for age, education and sex.

|  | **Age** | | | | | | |  |
| --- | --- | --- | --- | --- | --- | --- | --- | --- |
| **Education** | **35≤** | **36-45** | **46-55** | **56-65** | **66-75** | **76-80** | **≥81** | **Total** |
| **5≤** | 0/0 | 0/0 | 1/1 | 0/0 | 2/4 | 3/6 | 1/8 | 7/19 |
| **6-8** | 1/0 | 3/2 | 5/3 | 5/3 | 2/10 | 3/8 | 3/6 | 22/32 |
| **9-13** | 10/4 | 8/10 | 14/40 | 34/54 | 10/27 | 5/3 | 2/2 | 83/140 |
| **14-16** | 5/8 | 0/3 | 5/13 | 5/5 | 7/6 | 0/0 | 0/0 | 22/35 |
| **≥17** | 5/15 | 0/4 | 8/19 | 15/19 | 8/5 | 5/3 | 1/1 | 42/66 |
| **Total** | 21/27 | 11/19 | 33/76 | 59/81 | 29/52 | 16/20 | 7/17 | 176/292 |

**Notes.** Each cell shows the number of male/females.
